# Genetic Association of ICAM-1 (rs5498) Gene Polymorphism With Susceptibility to Stage II Grade B Periodontitis: A Case-Control Study in South Indian Population

**DOI:** 10.7759/cureus.56629

**Published:** 2024-03-21

**Authors:** Devika Bajpai, Arvina Rajasekar

**Affiliations:** 1 Periodontics, Saveetha Dental College and Hospitals, Saveetha Institute of Medical and Technical Sciences, Saveetha University, Chennai, IND

**Keywords:** genetic association, alleles, gene polymorphism, icam-1, periodontitis

## Abstract

Introduction: In the contemporary perspective, periodontitis is considered a complex issue triggered and perpetuated by bacteria but strongly influenced by the way the body reacts to bacterial plaque. Recent research has indicated that variations in genes might have an impact on the development of periodontitis. This study was conducted to explore a probable link between the genetic variations in intercellular adhesion molecule-1 (*ICAM-1*) represented by rs5498 and the occurrence of periodontitis.

Methods: A total of 100 participants, 50 with periodontitis and 50 with periodontally healthy or mild gingivitis, were recruited for this study. Whole blood drawn from the participants was used to obtain genomic DNA. The *ICAM-1 *gene polymorphism (rs5498) was determined using polymerase chain reaction (PCR) amplification and digestion. The *ICAM-1* gene's flanking primers were used to amp up the DNA. For statistical analysis, the genotype that was analyzed using the pattern of restriction fragment length polymorphism was recorded. The Chi-square test compared genotype and allele frequency distributions between both groups. The odds ratio with 95% confidence intervals with each individual allele or genotype was used to compute the risk. Statistical significance was established in all tests when the p-value was less than 0.05.

Results: There was no discernible difference between the genotype frequencies of patients and controls χ2df (P = 0.6065). The findings demonstrated that no significant difference was present between the two groups for homozygous or heterozygous mutant genotypes (AA vs. AG+GG; P = 0.6854). There was no discernible difference in the detected frequencies of the A allele (58% vs. 61%), G allele (42% vs. 39%), TT (16% vs. 24%), AG (40% vs. 36%), and TT genotypes in the studied groups.

Conclusion: According to the results of the current investigation, the *ICAM-1 *(rs5498) gene polymorphism is not associated with periodontitis in the population investigated.

## Introduction

Periodontal disease is an infectious condition that involves the supporting tissues of the tooth and is brought on by intricate interactions between the human immune system and the microorganisms in plaque. It is one of the illnesses that affects people the most frequently. However, only a small portion of patients get periodontitis, which is characterized by irreversible periodontal tissue damage. Numerous risk factors have been linked to the onset and development of periodontitis, but the specific susceptibility of patients to the disease has remained a mystery. These determinants include the microbiological makeup of plaque on teeth, subject features, social and behavioral aspects, and systemic and genetic aspects.

In the past, it was thought that everyone was equally susceptible to periodontal disease and that plaque buildup, poor dental hygiene, and possibly occlusal trauma were all that were required to cause periodontitis. However, during the past 40 years, it has come to be recognized that certain bacterial infections constitute the root of periodontal disease and that no two people are equally susceptible to either the infections or the harm they might inflict. This knowledge has led the experts to focus on the development of markers that will enable the identification of individuals who are susceptible before they acquire periodontitis and the identification of risk factors that may be adjusted in order to prevent or alter the course of periodontal disease. Investigations into periodontal disease susceptibility have gained more significance as a result of the understanding of potential connections between periodontal disease and systemic health that has arisen over the past ten years [1].

Our understanding of a variety of chronic immuno-inflammatory disorders, including periodontal disease, has recently taken on new significance as a result of one of biology's greatest achievements: the sequencing of the human genome. This has opened up fresh avenues for medical and dental research. The number of findings linking genes, genetic polymorphisms, and the development of periodontal disease is increasing exponentially. Inflammatory and immunological reactions in periodontitis are known to be influenced by genetic factors. Researchers have focused on finding genetic variations in many elements of immunity since the immune system is critical in the etiology of periodontitis. Numerous gene loci's allelic variations likely affect a person's susceptibility to periodontitis. While some of these genetic variations may have major and clinically relevant impacts, others are more likely to have small or insignificant effects [2].

The Ig superfamily includes intercellular adhesion molecule-1 (*ICAM-1*) [3]. Leucocytes, endothelial cells, monocytes, synovial cells, fibroblasts, and epithelial cells, all express *ICAM-1 *to varying degrees [4]. *ICAM-1 *can encourage cell attachment and draw leucocytes during inflammatory and immune responses. These *ICAM-1 *properties have been linked to rheumatoid arthritis [5]. According to one study, the *ICAM-1* levels in plasma and synovial fluid were significantly greater in rheumatoid arthritis patients compared to normal healthy controls [6]. *ICAM-1 *is a gene found at locus 19p13.3-p13.2 [7]. *ICAM-1* facilitates leukocyte adherence to the blood artery wall, allowing leukocytes to penetrate the tissues via transendothelial migration as an essential component of the initial immune response [8].

The immune system of the host may be impacted by *ICAM-1 *gene polymorphisms. Exon 6 of the *ICAM-1* gene promoter has the polymorphism rs5498 T > C, which has been linked to numerous illnesses, including atherosclerosis, myocardial infarction, coronary artery disease, and stenosis [9]. In this study, the role of *ICAM-1 *gene polymorphisms in the susceptibility to periodontal diseases was discussed and also the associations between the two groups in the South Indian population were studied because the disease progression of periodontitis is similar to that of other chronic inflammatory diseases. There is no evidence that a case-control study has been conducted correlating *ICAM-1* gene polymorphisms and chronic periodontitis [10].

## Materials and methods

This study conforms to the Helsinki Declaration. The Institutional Human Ethical Committee of Saveetha Dental College gave approval number IHEC/SDC/PERIO-2101/23/319. This cross-sectional study included 100 patients who reported to the Department of Periodontics, Saveetha Dental College and Hospitals, Chennai, India. Based on the clinical evaluation of the probing pocket depth (PPD), clinical attachment loss (CAL), and bleeding on probing (BOP), the individuals were split into two groups: group A, the periodontitis group, and group B, the control group. Both groups contained 50 patients. The periodontitis patients were identified according to the American Association of Periodontology (AAP) 2018 classification [11]. Individuals included in this study were systematically healthy with stage II grade B periodontitis or above whereas pregnant women, smokers, people with impaired immune systems, and participants who had recently received periodontal therapy were all disqualified from this study.

Sample collection 

The antecubital fossa was used to collect 2 ml of venous blood and transferred to a sterile tube containing ethylene diamine tetra acetic acid. It was blended carefully to prevent the formation of a clot. DNA isolation was carried out according to a modified Miller et al. 1988 technique [12].

Polymerase chain reaction and restriction endonuclease digestion

**Figure 1:**
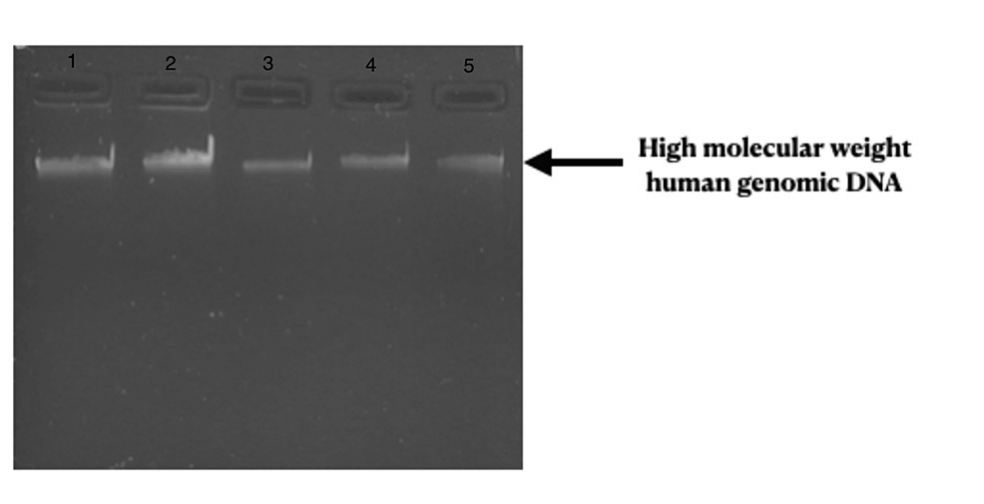
High molecular weight human genomic DNA High molecular weight human genomic DNA isolated from peripheral blood samples. The well contains genotypes 1: AA, 2: AG, 3: GG, 4: A, 5: G

**Figure 2:**
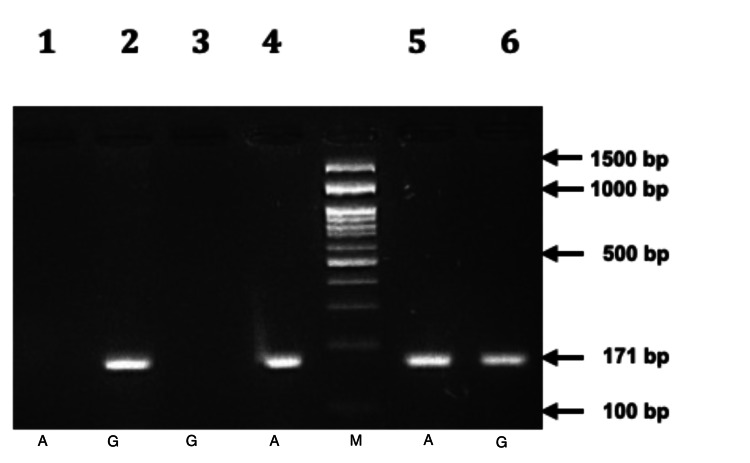
Examination of the PCR product using agarose gel A/G polymorphism of the ICAM-1 gene (rs5498): Allele-specific PCR amplification (171 bp) illustrating the various genotypes (Lane M = 100 bp DNA marker). Lane 1 and 2, G allele-specific primer amplification, indicating GG homozygosity (variant). Lane 3 and 4, A allele-specific primer amplification indicating AA homozygosity (wild type). Lane 5 and 6, both A and G allele-specific primers amplification, indicating AG heterozygosity.

Polymerase chain reaction (PCR) amplification and digestion were used to analyze the ICAM-1 gene polymorphism (rs5498). For DNA amplification, the following primers were used: forward primer: 5'-CTCAAGGGGAGGTCACCCGCA-3' and reverse primer: 5'-GCGGCTGCTACCACAGTGATG-3'. Then 10 nanograms of DNA were added in 5 pmol/μl of primer (forward and reverse), and PCR master mix (Takara Bio Inc., Shiga, Japan) was used to amplify DNA in 20 microlitre volume. The cycling conditions were as follows: initial denaturation took place at 94 °C for five minutes, followed by denaturation for 35 seconds at 94 °C, annealing for 25 seconds at 64 °C, extension for 35 seconds at 72 °C, and a final extension for five minutes at 72 °C. On a 2% agarose gel, high molecular genomic DNA was isolated from peripheral blood samples (Figure 1). A 2.5% agarose gel was used to visualize the digested product, and the results were documented (Figure 2).

Statistical analysis

IBM SPSS Statistics for Windows, Version 23.0 (Released 2015; IBM Corp., Armonk, New York, United States) was used for statistical analyses. The chi-square test was done to compare genotype and allele frequency distributions between the periodontitis and control groups. The odds ratio with 95% confidence intervals was used to calculate the risk related to specific alleles or genotypes. P < 0.05 was used to determine statistical significance.

## Results

The mean age for groups A and B was 38.04 ± 8.12 and 40.36 ± 7.45, respectively. The clinical parameters were assessed for both groups (Table 1). The genotype and occurrence rate of different genotypes were determined (Tables 2, 3). There was no difference in the rate of occurrence of genotypes between both groups χ 2df (p = 0.6065). This study demonstrated that no statistical difference was found between AA and AG + GG, p = 0.6854. The frequencies of AG and GG were found to be (40%; 36%) and (16%; 24%), respectively, and showed no significant difference. No statistical difference was seen in alleles A (58%; 61%) and G (42%; 39%); p = 0.6657.

**Table 1:** Demographic characteristics of the study population CAL, clinical attachment level; PPD, pocket probing depth; Gl, gingival index

Characteristics	Group A	Group B
Male	24	27
Female	26	23
Total	50	50
Mean age	38.04±8.12	40.36±7.45
CAL	6.15±1.25	-
PPD	5.86±1.17	1.65±0.54
GI	1.78±0.24	0.74±0.14

**Table 2:** The occurrence rates of different genotypes for the ICAM-1 (rs5498) gene polymorphism in both groups.

Groups	AA	AG	GG	A	G	HWE (p-value)
Cases (n=50)	20	18	12	0.58	0.42	0.064
Control (n=50)	22	20	8	0.61	0.39	0.154

**Table 3:** The distribution of genetic makeup of ICAM-1 (rs5498) gene polymorphism among both groups. A chi-square statistical test was performed to obtain p-values. OR, odds ratio; CI, confidence interval

Trait	Genotype	Case	Control	Unadjusted OR (95% Cl)	p-value
Dominant	AA	20	22	0.8485 [0.382-1.8788]	0.6854
	AA+GG	30	28		
Recessive	AG+AA	38	42	0.6032 [0.2227-1.6338	0.3200
	GG	12	8		
Allele	A	58	61	0.8829 [0.5018-1.5534	0.6657
	G	42	39		

## Discussion

Periodontitis is a multifaceted and intricate ailment resulting from a mix of genetic and environmental elements. In addition to pathogenic bacteria and various environmental factors such as smoking and stress that contribute to periodontitis, evidence indicates that genetic factors may also play a role in its onset [13]. Exploring genetic allelic variations has become increasingly important in assessing patients’ risk of developing periodontal diseases [14]. Recent studies on ICAM-1 gene polymorphisms have revealed associations with various chronic systemic illnesses [15].

Based on our investigation's findings, no statistically significant distinctions were observed in the genotype frequencies and distributions of the ICAM-1 (rs5498) polymorphism, as evidenced by a chi-squared test with χ 2df (p = 0.6065). Furthermore, our study revealed no noteworthy variations between the periodontitis and control groups concerning the homozygous or heterozygous mutant genotypes (AA vs. AG + GG; p = 0.6854). Specifically, the frequency of TT (16% vs. 24%) and AG (40% vs. 36%) genotypes did not display any noticeable differences between the periodontitis group and control groups. Additionally, the G allele (42% vs. 39%) and A allele (58% vs. 61%) frequencies were not found to be significantly different between the periodontitis and control groups.

The inactive hepatocyte growth factor (HGF) triggers the expression of ICAM-1 mRNA [16]. Moreover, pro-inflammatory cytokines, like IL-1b, TNF-a, IFN-g, and IL-2, can also stimulate ICAM-1 [17]. ICAM-1 serves as an adhesion receptor on both leukocytes and endothelial cells, facilitating the movement of inflammatory cells into the human gingival epithelium [18]. Previous research suggests that ICAM-1 might play a crucial role in the development and advancement of chronic periodontitis [19]. These findings imply that genetic variations in ICAM-1 (single nucleotide polymorphisms, SNPs) could influence susceptibility to periodontal issues, potentially impacting the severity of periodontitis based on these variations. By identifying ICAM-1 polymorphisms, it may be feasible to intervene in the context of periodontitis, potentially curbing its progression and enabling targeted treatments at the genetic level.

ICAM-1 rs5498 was linked to chronic periodontitis in the Heilongjiang Chinese population according to a prior study by Wang et al. [20]. Earlier the investigation by Sun et al., which opened the door for the current study, showed that polymorphisms and protein levels of the ICAM-1 gene may be associated with periodontitis and alter its progression [21]. However, according to the study’s findings, the ICAM-1 gene polymorphism and periodontitis did not appear to be significantly related. Similar to other chronic diseases, the pathophysiology of periodontitis is characterized by a number of cellular processes that ultimately result in the same clinical manifestation [22].

Limitation

The limitations of this study are the small population size and varied ethnic composition, which may explain any discrepancies in the findings. It is significant to remember that different ethnic populations may have different numbers and types of genes responsible for similar diseases. A functional SNP might therefore be in linkage disequilibrium with several markers in various ethnic groups.

## Conclusions

The current research indicates that there is no connection between the ICAM-1 (rs5498) gene polymorphism and periodontitis within the group under examination. Additional investigations are needed to investigate how the ICAM-1 gene interacts with epigenetic factors in the development of periodontitis and its potential association with systemic diseases among periodontitis patients.
